# Small Molecule Inhibitors of the LEDGF Site of Human Immunodeficiency Virus Integrase Identified by Fragment Screening and Structure Based Design

**DOI:** 10.1371/journal.pone.0040147

**Published:** 2012-07-10

**Authors:** Thomas S. Peat, David I. Rhodes, Nick Vandegraaff, Giang Le, Jessica A. Smith, Lisa J. Clark, Eric D. Jones, Jonathan A. V. Coates, Neeranat Thienthong, Janet Newman, Olan Dolezal, Roger Mulder, John H. Ryan, G. Paul Savage, Craig L. Francis, John J. Deadman

**Affiliations:** 1 CSIRO Materials, Science and Engineering, Parkville, Victoria, Australia; 2 Avexa Ltd, Melbourne, Victoria, Australia; 3 CSIRO Materials, Science and Engineering, Clayton, Victoria, Australia; Duke University Medical Center, United States of America

## Abstract

A fragment-based screen against human immunodeficiency virus type 1 (HIV) integrase led to a number of compounds that bound to the lens epithelium derived growth factor (LEDGF) binding site of the integrase catalytic core domain. We determined the crystallographic structures of complexes of the HIV integrase catalytic core domain for 10 of these compounds and quantitated the binding by surface plasmon resonance. We demonstrate that the compounds inhibit the interaction of LEDGF with HIV integrase in a proximity AlphaScreen assay, an assay for the LEDGF enhancement of HIV integrase strand transfer and in a cell based assay. The compounds identified represent a potential framework for the development of a new series of HIV integrase inhibitors that do not bind to the catalytic site of the enzyme.

## Introduction

Highly active antiretroviral therapies (HAART) have been an effective way to slow the spread of HIV, but the virus continues to develop resistance to all drugs that have been used in treatment to date [1]. HIV integrase (IN) is one of the viral targets for which small molecule therapeutics have now been approved and marketed to treat AIDS [2]. IN is a critical enzyme in the HIV life cycle as it is required for the integration of viral DNA into the host chromatin, and therefore persistence of infection. IN performs both the 3′ viral processing step as well as the strand transfer step to insert viral DNA into the host DNA. The structure of HIV IN has been thoroughly investigated [3]–[7], and consists of three domains (N-terminal DNA binding, catalytic core (CCD) and C-terminal DNA binding). The CCD has several pockets to which small molecules have been shown to bind and inhibit the enzymatic activity [6], [8]–[12]. There are several inhibitors currently used in therapy or in late stage trials that target the catalytic site of HIV IN, making it a validated and attractive target for new therapies. IN forms a complex with viral DNA and several host cellular factors that has been termed the pre-integration complex (PIC) [13]. One component of this complex is lens epithelium derived growth factor (LEDGF/p75), which has a conserved IN binding domain (IBD, residues 347–429) that mediates binding [14]. Within the IBD is a loop (residues 362 to 369) that binds a pocket formed by a dimer of the HIV IN CCD. Small peptides composed of the residues in this IBD loop can bind to the IN CCD with micromolar affinity and can compete with the IBD for this binding site [15]. Several crystal structures with various peptide sequences have been solved showing the interactions between these peptides and IN [16]. In addition, full length IN and LEDGF make further interactions through the N-terminal domain of IN [17], [18]. Furthermore, an epidemiological study of polymorphisms in the *PSIP1* gene that codes for the LEDGF protein has shown variation in serologic levels of HIV virus and pathogenesis, consistent with the role of LEDGF in disease progression [19], [20].

LEDGF promotes the localization of IN to the host chromosome, and it has been shown that the PWWP domain of LEDGF plays an essential role in localising the site of integration to genes, such that a PWWP domain deletion mutant leads to loss of interaction with condensed chromatin [21]–[23]. An *in vitro* assay estimated the enhancement of IN binding to DNA by LEDGF to be 30 to 50 fold [24]. Therefore disrupting the interaction of LEDGF and HIV IN appears attractive for therapeutic purposes. In addition, the LEDGF binding site of HIV IN is well conserved, making it a tantalizing target for inhibitors [11], [14], [18], [19]. While our study was underway, several groups reported small molecule inhibitors that bind to this site in HIV IN [8], [25]–[28].

One group used a pharmacophore based on the interactions of the residues of the IBD (I365, D366 and L368) to give a lead compound CIBA-3003 which inhibited the LEDGF interaction with IN in an AlphaScreen (AS) assay with an IC_50_ of 35 µM [25].

Starting from a catechol scaffold that is well known to chelate metals in the active site of HIV IN, a series of benzamides were synthesised and led to *N*-(cyclohexylmethyl)-2,3-dihydroxy-5-(piperidin-1-ylsulfonyl) benzamide, that had an IC_50_ of 8 µM and CC_50_>40 µM [26]. Recently, activity against infection of whole cells was reported and this compound also bound at the active site and inhibited strand transfer with an IC_50_ of 19 µM.

A pharmacophore was also used to screen a virtual set of 160,000 compounds and based on further docking results, 25 compounds were purchased and assayed in an AS assay. This identified 2-(quinolin-3yl)acetic acids (LEDGINs) as a lead series. Further studies led to an inhibitor (LEDGIN-6 [8]) with an AS IC_50_ of 1.37 µM and an EC_50_ of 2.73 or 3.45 µM for HIV-1 infection of MT-4 or PBMC cells, respectively. This compound was crystallised with the Phe185Lys mutant of the CCD domain of HIV IN and shown to form hydrogen bonds with residues Glu170, His171 and Thr174 of HIV IN (PDB: 3LPU) [8]. However this compound was also found to have an IC_50_ of 19.5 µM for inhibition of the strand transfer activity of HIV IN, suggesting that it could either bind at the active site or be an allosteric inhibitor, as recently reported [11], [12]. Further iteration led to a thieno[2,3-b]pyridine series, of which the lead had an IC_50_ 0.58 µM in MT-4 cells, an EC_50_ 0.76 µM and a CC_50_ of 72.1 µM. Also, further testing of these inhibitors using known mutations of HIV IN, Ala128Thr and in HIV-2 Ala128Met, showed more than 100 fold resistance to these compounds, suggesting the existence of interactions in addition to those of amino acids 170, 171 and 174 [8]. A second compound, CHI-1043, returned an IC_50_ of 0.14 µM in the active site directed strand transfer assay, but only 36 µM in the AS assay for binding at the LEDGF site [27].

However, of the above studies, only two used crystallography to identify the exact binding mode of similar quinoline based core compounds [8], [12], whereas the others relied on molecular modelling to infer which groups were important for interaction at either site.

The study of cyclic peptides bound to the LEDGF site [16] show hydrogen bonding interactions with Gln168 (e.g. PDB 3AVB), while the small molecules reported to date do not make this interaction. We herein present a novel, small molecule inhibitor of HIV IN that binds to the LEDGF site, discovered through fragment based screening and structure based drug design, which has low micromolar activity in both biochemical and cellular infectivity assays.

## Materials and Methods

### Protein Expression and Purification

N-terminally hexa-His tagged catalytic core domain (CCD) IN (residues 50 to 210) containing the mutations C56S, F139D and F185H (core3H) was cloned into the *E. coli* expression vector pET28b(+) (Novagen) and expressed and purified essentially as described for core4H [9] (which has an additional W131D mutation), however the current studies retained the N-terminal His tag.

Hexa-His tagged full length HIV IN was expressed and purified as follows: full length HIV IN was amplified using an N-terminal primer introducing an Nde1 site at the N-terminus and a C-terminal primer introducing an Xho1 site following the HIV IN termination codon. The product was purified, digested with Nde1 and Xho1 and cloned into the pET28b(+) (Novagen, Merck KgGA, Darmstadt, Germany) expression vector. Sequencing of the clone confirmed a sequence identical to the NL4-3 IN sequence. An overnight culture of transformed BL21(DE3) cells grown in TB kanamycin (30 ug/ml) was diluted 1 in 100 into fresh TB kan and grown at 30°C. When an OD600 of 0.6 was reached, IPTG was added to 1 mM and cultures induced at 30°C for 6 hours. Cells were then lysed on ice for 30 min in 25 mM HEPES, pH 7.5, 2 mM β-mercaptoethanol (BME), 5 mM imidazole, 0.3 mg/ml lysozyme followed by the addition of Omnicleave (20 U/ml) (Epicentre, Madison, WI) and 2 mM MgCl_2_ for an additional 30 min at room temperature. Cell debris was pelleted (20,000 g, 20 min), and the pellet was extracted twice with 25 mM HEPES pH 7.5, 0.5 M NaCl, 5 mM BME, 10 mM imidazole, 5% (v/v) glycerol. The supernatant was discarded and the pellet extracted an additional two times with Buffer A (25 mM HEPES, pH 7.5, 1.5 M NaCl, 5 mM BME, 10 mM imidazole, 10% (v/v) glycerol). This supernatant was adjusted to 1 M NaCl with 25 mM HEPES pH 7.5, and applied to a 5 mL immobilised metal affinity HiTrap column (GE Healthcare, Uppsala, Sweden) charged with nickel and equilibrated with buffer A containing 1 M NaCl. The column was washed with 10 volumes of Buffer A containing 1 M NaCl and 60 mM imidazole (buffer B) and eluted with a linear gradient of Buffer B (Buffer B containing 1 M imidazole). The highly purified IN containing fractions were pooled and dialysed against 25 mM Tris-Cl pH 7.5, 0.5 M NaCl, 40% (v/v) glycerol, 5 mM DTT then snap frozen in liquid nitrogen and stored at −80°C.

A FLAG-His_6_ control fusion protein (comprised of MDYKDDDDKS-LEDGF amino acids^1–15^-RAALEHHHHHH) was cloned into pET28b(+) and expressed in *E. coli* and purified by Ni affinity chromatography as described for hexa-His tagged full length HIV IN.

A FLAG epitope tagged LEDGF protein was prepared by fusing the FLAG tag (DYKDDDDK) to the N-terminus of the LEDGF protein. LEDGF protein was purified as described by Maertens et al [17] with the following modifications: *E. coli* BL21(DE3) cells containing FLAG-LEDGF cloned into pET28b(+) were grown in LB until OD600 of 0.9 and expression was induced at 30°C for 6 hours. Cells were pelleted and resuspended in 30 mM Tris-HCl, pH 7.0, 450 mM NaCl, 1 mM DTT, 1 mM EDTA, 1 mM PMSF and lysozyme (0.3 mg/ml), Omincleave (20 U/ml) and MgCl_2_ (2 mM) added. The cell pellet was extracted on ice for 20 min with shaking. Following centrifugation at 15000 g for 20 min, the supernatant was diluted to reduce the salt concentration to 300 mM NaCl and applied to a Fast Flow 5 mL Heparin column (GE Healthcare, Uppsala, Sweden) equilibrated in 30 mM Tris-HCl, pH 7.0, 300 mM NaCl, 1 mM DTT, 1 mM EDTA. The column was washed with 30 mM Tris-HCl, pH 7.0, 300 mM NaCl, 5 mM BME, 2.5 mM CHAPS and FLAG-LEDGF was eluted with a linear gradient of 30 mM Tris-HCl, pH 7.0, 1.5 M NaCl, 5 mM BME, 2.5 mM CHAPS. The fractions containing FLAG-LEDGF (eluted at approx. 1 M NaCl) were pooled, diluted to give a NaCl concentration of 50 mM and applied to an SP column in 30 mM Tris-Cl pH 7.0, 50 mM NaCl, 5 mM BME, 5% (v/v) glycerol, washed and eluted with a linear gradient in the same buffer but containing 1 M NaCl. The presence of the FLAG-LEDGF construct was confirmed by Western blot using an anti-FLAG-alkaline phosphatase antibody (SigmaAldrich, Australia). Fractions containing LEDGF were pooled, dialysed against 25 mM Tris-Cl pH 7.0, 0.5 M NaCl, 5 mM DTT, 5% (v/v) glycerol, snap frozen and stored at −80°C. Untagged LEDGF was expressed in and purified from *E. coli* as described for FLAG-LEDGF.

### AlphaScreen and Counter Screen

For the IN-LEDGF AS assay, compound dilutions and His_6_-tagged IN (30 nM final concentration) were initially incubated together in 10 µl of reaction buffer (25 mM Tris-Cl pH 7.5, 25 mM NaCl, 5 mM MnCl_2_, 5 mM MgCl_2_, 5 mM BME, 50 µg/ml bovine serum albumin and 0.05% (v/v) Tween-20) for 10 min at room temperature in AlphaPlate™-384 SW plates (Perkin-Elmer). Subsequently, 10 µl reaction buffer containing Flag-tagged LEDGF (30 nM final concentration), AS Ni-chelate donor beads (Perkin-Elmer; 5 µg/ml final) and AlphaLISA anti-FLAG acceptor beads (Perkin-Elmer; 5 µg/ml final) were added and protein association allowed to occur for 1 hour in the dark at 30°C before emission from the donor beads was measured using a PHERAstar FS plate-reader (BMG Labtech). Counter screen assays to ensure the compounds were not inhibiting the binding of the His or FLAG tags to the beads or quenching of the signal, were performed under the same reaction conditions except that a Flag-His_6_ fusion protein was used (20 nM final) instead of individual His_6_-tagged IN and Flag-tagged LEDGF proteins, and the initial 10 min incubation at room temperature was omitted. The specificity of FLAG-LEDGF for IN binding was confirmed by competing the FLAG-LEDGF with untagged LEDGF. At a 1∶1 molar ratio with FLAG-LEDGF, untagged LEDGF reduced the AS signal by 50%.

### Virus Infectivity Assay

HuT78 cells were seeded into 96 well microtiter plates at 50,000 cells per 50 µl per well in RF-10 containing 2 µg/ml polybrene (RF-10/2). Compounds were prepared to 4× final concentration in RF-10/2, and 30 µl was added to cells. HIV-1_NL4–3_ (40 µl in RF-10/2 containing 1600 plaque forming units (pfu)) was added to each well or 40 µl of RF-10/2 was added to the negative controls, and when assaying compound cytotoxicity. pfu were determined by titrating virus stocks on HT4LacZ cells as described by Rocancourt et al [29]. After 24 hours, an additional 90 µl of media or media containing 1× compound was added to each well. At 4 days post-infection, 100 µl of media was removed from each well and replaced with 100 µl of fresh media with or without compound. 48 hours later supernatants were harvested and levels of extracellular p24 determined. Supernatants were diluted 1 in 10,000 and p24 levels assayed using the Vironostika p24 assay kit. EC_50_ was calculated as the concentration required to inhibit HIV p24 production to 50%.

### Activity Against IN with QHGS or NHEQ Mutations at the Active Site

Single-round infectivity assays were performed with 293T cells. The assays used virus stocks generated by transfecting a plasmid encoding a modified full-length HIV-1 genome together with an expression plasmid producing the vesicular stomatitis virus envelope glycoprotein (VSV-G) [30]. The modified HIV-1 genome used in the assays had the envelope gene deleted and had a reporter luciferase gene cloned into the nef region (pHIV env-Luc). Culture supernatants containing VSV-G pseudotyped virions were harvested 64 hours post-transfection, clarified by centrifugation to remove cell debris, and stored at −80°C. HIV IN mutations known to confer resistance to published IN inhibitors were prepared by site directed mutagenesis of the pol (IN) gene in a shuttle vector which contained the majority of the HIV gag and pol sequence. The mutated IN coding region was then sequence verified and exchanged for the wild-type coding sequence in the pHIV env-Luc reporter virus plasmid. Single round infection assays were conducted in 293T cells seeded in 96 well plates 16 hours prior to compound addition. Compounds were pre-incubated with cells for 4 hours at 37°C followed by the addition of sufficient virus to generate approximately 10,000 Luciferase light units in the absence of compound. Luciferase readout was measured 48 hours post-infection using the Bright-Glo™ reagent (Promega) according to the manufacturer’s instructions.

### SPR Measurements of Analogue Compounds to Explore Structure Activity Relationships

Core3H and core4H proteins (∼3×10^−8^ moles) were “minimally” biotinylated [31] on ice for 2 hours using an equimolar concentration of EZ-Link® Sulfo-NHS-LC-LC-Biotin (Pierce). To remove unreacted biotin reagent, biotinylated proteins were passed through a Superdex 75 (10/300 GL) column equilibrated in ‘SPR capture’ buffer (50 mM HEPES pH 7.4, 150 mM NaCl, 0.05% (v/v) Tween20, 5 mM DTT, 10 mM MgCl_2_). The eluted proteins were stored frozen in 50 µl aliquots at −70°C. Streptavidin (Sigma) was immobilized at 25°C onto a CM7 sensor chip (GE Healthcare) docked in a Biacore T200 upgrade instrument (GE Healthcare) using a previously described procedure [32] resulting in immobilization levels of approximately 12000 RU of protein being coupled in all flow cells. The biotinylated core IN proteins were captured onto a Streptavidin chip surface in SPR capture buffer by injecting at 5 µL/min for 5 min over a single flow cell, typically resulting in immobilization of approximately 2300 RU of target protein. A minimally-biotinylated tyrosine kinase domain of CSF-1-R was captured (∼4300 RU) in a separate flow cell to provide for an unrelated negative control. The blank (typically flow-cell 1) and protein captured surfaces were subsequently blocked with two 1 min injections of 1 µg/mL D-biotin (Sigma) at 5 µL/min. All SPR binding experiments were performed at 20°C in SPR binding buffer (50 mM HEPES pH 7.4, 150 mM NaCl, 0.05% (v/v) Tween20, 5 mM DTT, 10 mM MgCl_2_, 5% (v/v) DMSO). Small molecules stock solutions were serially diluted (2-fold) in SPR binding buffer and injected for 30 sec contact time at 60 µL/min and then allowed to dissociate for 60 sec. The entire concentration series for each compound was performed in triplicate.

### Data Processing

Raw sensorgram data were processed, solvent corrected and double referenced using Scrubber software (BioLogic Software, Australia). For easy comparison between data sets, all experiments were normalized using a normalization formula of Giannetti *et al* [33]. To determine the binding affinity (*K_D_*), responses at equilibrium for each analyte were fitted to a 1∶1 steady state affinity model available within Scrubber.

### Crystallization and Data Collection

The ability to reproducibly generate high quality CCD IN crystals allowed us to generate all complex structures by soaking in the compounds with preformed HIV IN CCD crystals. The protein was crystallized as described previously [10]. Briefly, purified hexa-His core3H protein was concentrated to 5.5 mg/ml in 40 mM Tris pH 8.0, 250 mM NaCl, 30 mM MgCl_2_ and 5 mM DTT. Drops were set up in SD-2 (IDEX Corp) sitting drop plates using a Phoenix robot (Art Robbins Industries) with 50 µl of crystallant in the reservoir and droplets consisting of 200 nl of the reservoir and 200 nl of the protein sample. Final crystallization conditions were: 100 mM sodium acetate at pH 5.0 to pH 5.5 and 1.6–2.0 M ammonium sulfate at 20°C. A cryo-solution was made up of 100 mM sodium acetate pH 5.5, 1.75 M ammonium sulfate, 25% (v/v) ethylene glycol and 5% (v/v) DMSO. 1.5 µl of cryo-solution was added to the crystal containing droplet, powdered compound was then added to this expanded drop and the crystallization plate was resealed. 24 to 48 hours later the crystals were taken to the MX1 or MX2 beamline at the Australian Synchrotron for data collection. MicroLoops from MiTeGen were used to gently remove the crystal from the drop and place it in the 100 K nitrogen stream. 181 frames of data, each one a 1° oscillation for one second of exposure, were taken for each crystal [34].

### Structure Solution and Crystallographic Refinement

All data were indexed with Mosflm [35], scaled with SCALA [36] and molecular replacement was done using Phaser [37]. Manual rebuilding was done with the molecular graphics program Coot [38]. Compounds were placed in density using Afitt (OpenEye Scientific Software) and 5 to 8 rounds of manual rebuilding and refinement (Refmac [39]) were done for each of the structures showing compound density. Figures showing the difference (Fo-Fc) density for compounds **2, 6, 8, 10**, and **11** can be found in the supporting information (Figure S1A–E).

### Synthesis of Compounds

Compounds **4**–**11** were designed at Avexa Ltd using the Accelrys Discovery suite and compounds **2–11** were prepared by the team of Dr Xian Bu at SYNthesis med-chem, Level 4, Buiding 21A, 528 Ruiqing Rd. Heqing, Pudong, Shanghai 201201.

## Results

**Figure 1:**
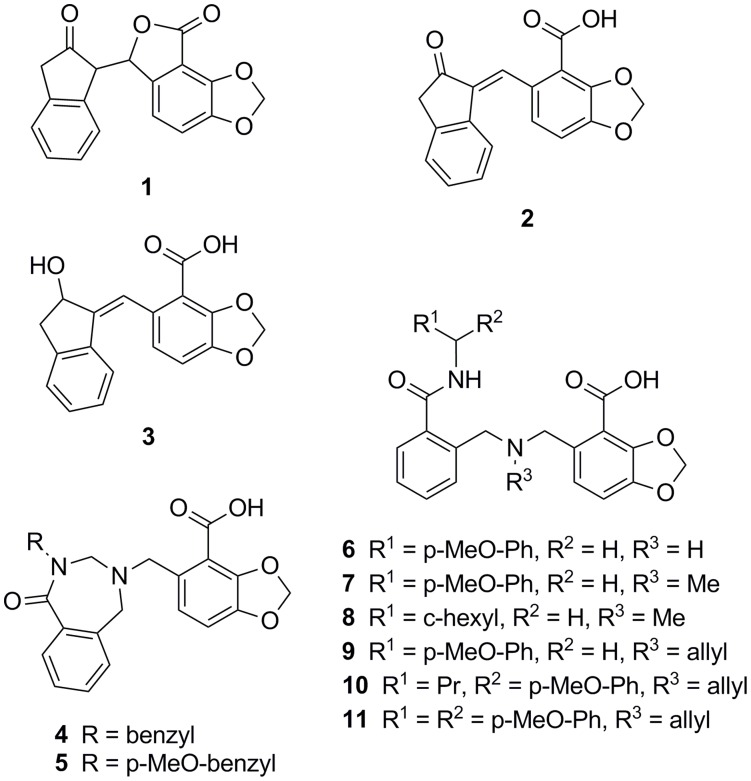
Chemical compounds explored to determine structure activity relationship with IN.

**Figure 2:**
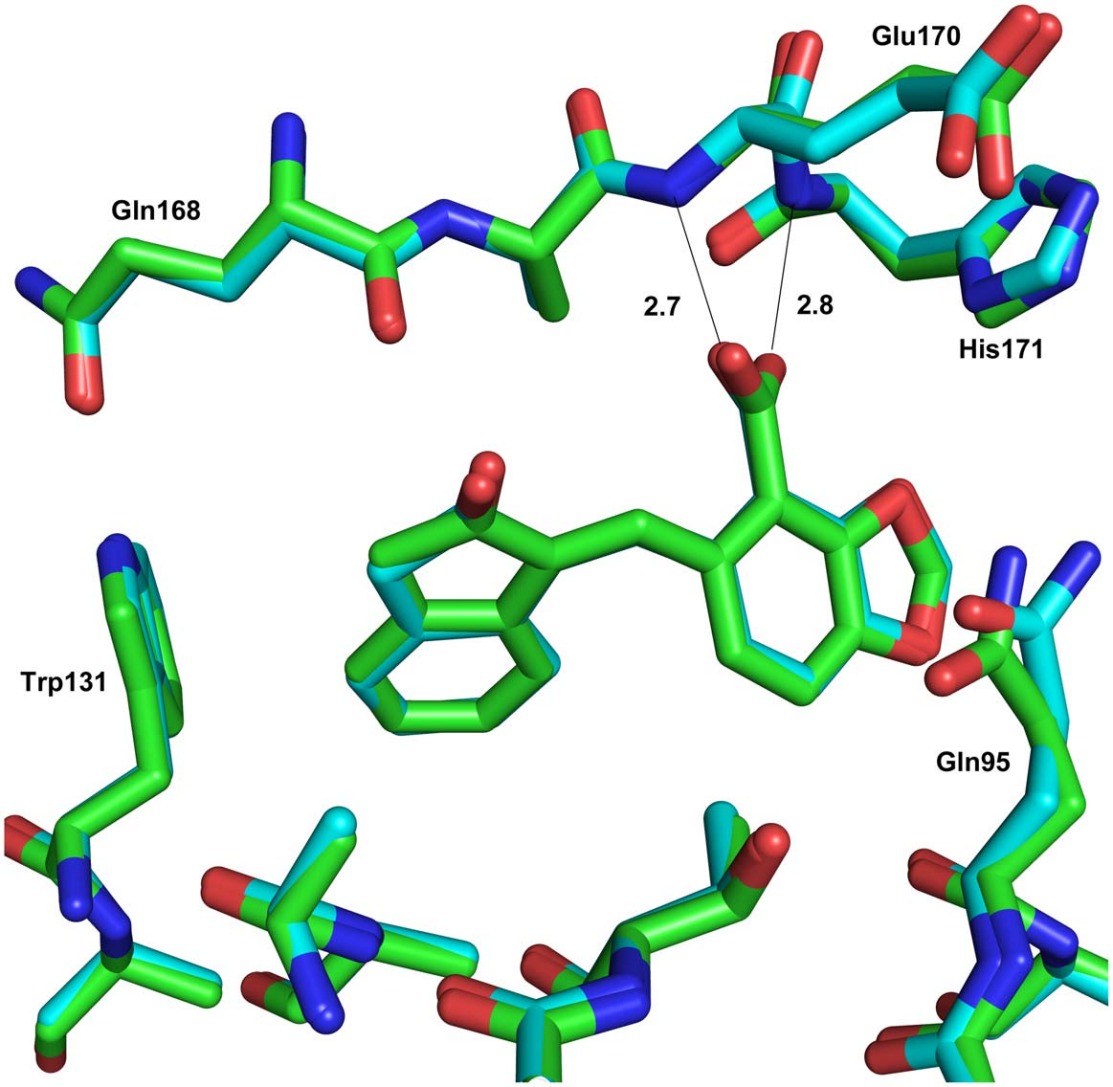
Overlay of structures in the LEDGF site. Obtained by soaking **1** (green) (PDB 3ZT4) and **2** (cyan) (PDB 3ZT3); hydrogen bond interactions between the backbone nitrogens of residues Glu170 and His171 and the compounds are noted. This figure shows that compounds **1** and **2** are identical in the crystal structures, despite compound **1** being in the ring closed form prior to addition to the integrase crystals. Superposition of the structures was done over the full protein sequence, and not just the LEDGF binding site, for all crystallographic figures.

We performed a fragment screen of 500 compounds using surface plasmon resonance (SPR), NMR and crystallography (data not shown) using the His-tagged IN-CCD protein from *E. coli*. This initial screen was done using a commercial library (Maybridge Ro3) via both SPR and NMR, and crystallographic analysis was used to confirm these initial hits. Based on the hits obtained from this screen, several analogues were chosen from the CSIRO compound library and these were tested via SPR and crystallography for binding affinity and the location of binding respectively. One of the hits that demonstrated good density in the LEDGF site was lactone **1**, which was found by SPR to have better affinity (750 µM vs 1570 µM, Table 1) for the core4H than the core3H construct of the CCD. Core4H has a Trp131Asp mutation not present in core3H, and this residue forms part of the wall of the LEDGF pocket [6], suggesting that this compound would not interact as well with wild type IN. Additionally it was found in the crystal and in assays that **1** existed as the ring-opened form and this form (compound **2**) was as active as the original sample in SPR assays (Figures 1 and 2, Table 1). The 1*H*-inden-2(3*H*)-one of **2** occupies the same position in the IN pocket (PDB 3ZT3, Table 2) as residue Ile365 in the LEDGF loop [6], [16]. In addition, the carboxylic acid of the compound makes a virtually identical interaction to Asp366 of the LEDGF loop [6], [16]. This charge interaction is key to the series developed here, to the series of peptides that have been shown to interact with the LEDGF binding site on HIV IN, and key for the binding of other small molecules that have been developed by other groups [8], [11], [12], [16], [25], [26], [28]. This was the basis for our first analogue **3**, which demonstrated similar affinity to **2** in the AS assay (AS) (270 µM and 200 µM respectively, Table 1), and the SPR core3H assay (1435 µM vs 1375 µM, Table 1) and clear density in the LEDGF site.

**Table 1:** All values in the table are in micromolar (µM).

Compound	AlphaScreen	Counter-Screen	PDBcode	SPRCore3H	SPRCore4H
**1**			3ZT4	1570±337	750±311
**2**	200		3ZT3	1375±149	763±372
**3**	270		3ZT2	1435±304	724±347
**4**	110		3ZT1	>2000	>2000
**5**	600		No	>2000	>2000
**6**	270		3ZSZ	>2000	>2000
**7**	220		3ZSY	595±16	1180±198
**8**	100		3ZSW	>2000	>2000
**9**	815		3ZSX	519±49	880±99
**10**	29	175	3ZSQ	10.9±2.7	74±16
**11**	8.1	145	3ZSO	7.6±1.2	76±11

Compound **1** was from the CSIRO library and was not tested in the AS, but the ring opened form (**2**) was synthesized and tested and showed the same structure and binding mode in the crystal structure.

**Table 2:** Data and model statistics for the 10 co-crystal complexes of compounds with HIV integrase.

Compound- PDB code	11-3ZSO	10-3ZSQ	9-3ZSX	8-3ZSW	7-3ZSY	6-3ZSZ	4-3ZT1	3-3ZT2	2-3ZT3	1-3ZT4
Spacegroup	P31	P31	P31	P31	P31	P31	P31	P31	P31	P31
Cell (Å) (a = b)×c	71.1×67.0	71.0×67.7	71.0×67.0	71.0×66.6	70.7×66.9	71.5×66.5	70.6×66.7	71.3×67.0	71.4×67.4	71.1×66.6
resolution (Å)	61.5–1.75	61.5–1.70	35.5–1.95	35.5–1.80	35.4–2.20	31.5–2.00	45.0–1.75	45.4–1.70	31.5–1.95	61.5–2.20
completeness (%)	99.9	99.9	99.7	100	99.9	100	100	98.5	99.9	100
Rwork % (high res bin)	16.3 (24.7)	16.1 (25.4)	18.4 (25.6)	17.4 (23.3)	18.1 (22.0)	18.7 (23.5)	17.5 (29.1)	17.9 (37.6)	17.2 (22.4)	17.1 (21.1)
Rfree % (high res bin)	20.9 (32.8)	20.0 (31.5)	22.8 (29.9)	20.6 (27.1)	22.2 (27.8)	22.2 (28.3)	19.9 (34.5)	21.6 (44.1)	21.6 (28.9)	23.2 (26.5)
# reflections	36193	39835	26077	33030	18024	24345	35588	39230	26560	18108
# atoms protein	2553	2468	2457	2483	2394	2407	2438	2407	2436	2433
# atoms ligand	146	116	126	122	102	109	117	138	98	134
# atoms solvent	166	225	126	123	68	96	121	126	119	103
Mean B value (Å^2^)	25.3	20.0	23.4	23.0	23.9	26.8	21.6	25.3	23.8	28.3
Ramachandran analysis (%) preferred/allowed/outliers	97.5/2.1/0.4	99.2/0.8/0	98.9/1.1/0	99.2/0.8/0	99.3/0.7/0	99.6/0.4/0	98.9/1.1/0	98.2/1.8/0	99.2/0.8/0	98.5/1.5/0

The compound number precedes the PDB (RCSB) code of the deposited structure. The numbers in parentheses are those for the highest resolution shell

(Rwork and Rfree).

Inspection suggested that a seven membered ring would more effectively fill this pocket and accordingly several 2,3,4,5-tetrahydro-1*H*-benzo[*e*] [1], [3]diazepin-1-one based analogues were synthesised (examples are compounds **4** and **5**). Several compounds in this series were active in the AS assay (e.g. compound **4** at 110 µM), but did not bind in the SPR assays. However, when **4** was tested in a virus infectivity assay the activity in the cell toxicity counter screen was of the same order of magnitude (275 µM and 265 µM, respectively), indicating that true activity could not be determined. This suggested a possible problem with cell permeability for this series. Upon analysis of the crystal of IN soaked with **5**, it was found that the ring-opened structure, compound **6** (a side product of the synthetic pathway) was bound (PDB 3ZSZ, Table 2). To confirm this result, compound **6** was isolated from the original preparative reaction mixture directly and this sample afforded the identical crystal complex and AS activity of 270 µM but binding could not be detected in the SPR assay.

**Figure 3:**
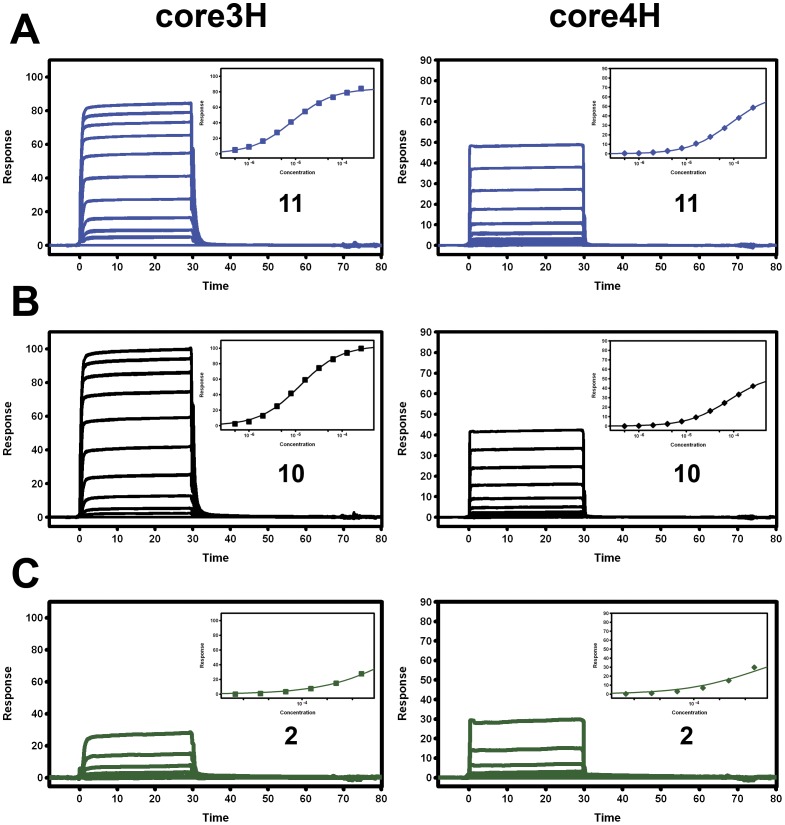
SPR sensorgrams showing three compounds binding to immobilized HIV integrase core3H (left panels) and core4H (right panels). (A) Compounds **11** and (B) **10** were injected as a ten-cycle, two-fold dilution series with a top concentration of 256 µM; compound **2** (C) was injected as a six-cycle, two-fold dilution series with a top concentration of 512 µM. Duplicate overlaid binding data are shown. Inset diagrams (top right in each panel) show the fit of the binding responses at equilibrium (t = 25–28, plotted against compound concentration) to a simple binding isotherm. Affinity estimates (K_D_ values) for these binding interactions are listed in Table 1. For compounds that failed to reach maximal binding response (R_max_) for top injected concentration (256 or 512 µM), K_D_ values were estimated using R_max_ values determined from binding fits obtained for **10**.

We noted that compound **6** contained a secondary amino group and subsequently several *N*-alkylated derivatives of **6** were prepared and encouragingly this modification restored binding in the SPR assay. Alkylation of the secondary amine of **6** led to **7** (R^3^ =  Me) which displayed similar levels of AS activity. Thus, tertiary amine **7** displayed an affinity of 595 µM in the SPR core3H compared to >2000 µM for secondary amine **6**. From **7,** replacement of the *p*-methoxyphenyl group led to **8**, (R^1^ =  cyclohexyl) which gave 100 µM inhibition in the AS activity assay but with loss of SPR activity, whereas **9** (R^1^ =  p-MeO-phenyl, R^3^ =  allyl) retained SPR activity. Analysis of the crystal complexes of compounds **6** to **9** suggested that branched amide analogues could more effectively fill the pocket, so we synthesized compounds **10** and **11.** Both had better activity in the AS assay, 29 µM and 8 µM respectively, with **11** having the best activity in the series. To confirm that compounds **10** and **11** were not giving a false positive reading in the AS assay, they were tested in a counter screen using the Flag-His6 fusion protein and showed respectively 6 and almost 20 fold less activity (175 µM and 145 µM, Table 1). Both compounds show the desired selectivity in the SPR assay for the core3H over the core4H CCD constructs (Table 1, Figure 3). A cell based HIV-1 infection assay was performed to obtain EC_50_ values, and **11** returned an EC_50_ of 29 µM and in the counter screen had a CC_50_ of >100 µM. To provide further evidence that compound **11** did not interact at the IN active site, the compound was assayed in the cell infectivity assay using IN double active site mutants, either Q148H/G140S (QHGS) or N155H/E92Q (NHEQ), and returned a similar EC_50_ of 54 µM (±4 SD) or 37 µM (±4 SD). In this same assay raltegravir [40] (which is the clinically approved IN inhibitor Isentress™) has an EC_50_ of 10 nM for the virus with the wild type IN, but was essentially inactive (EC_50_>1 µM) for virus with either of the QHGS or NHEQ mutations. Raltegravir has been confirmed to bind at the active site of IN by crystallography [41].

**Figure 4:**
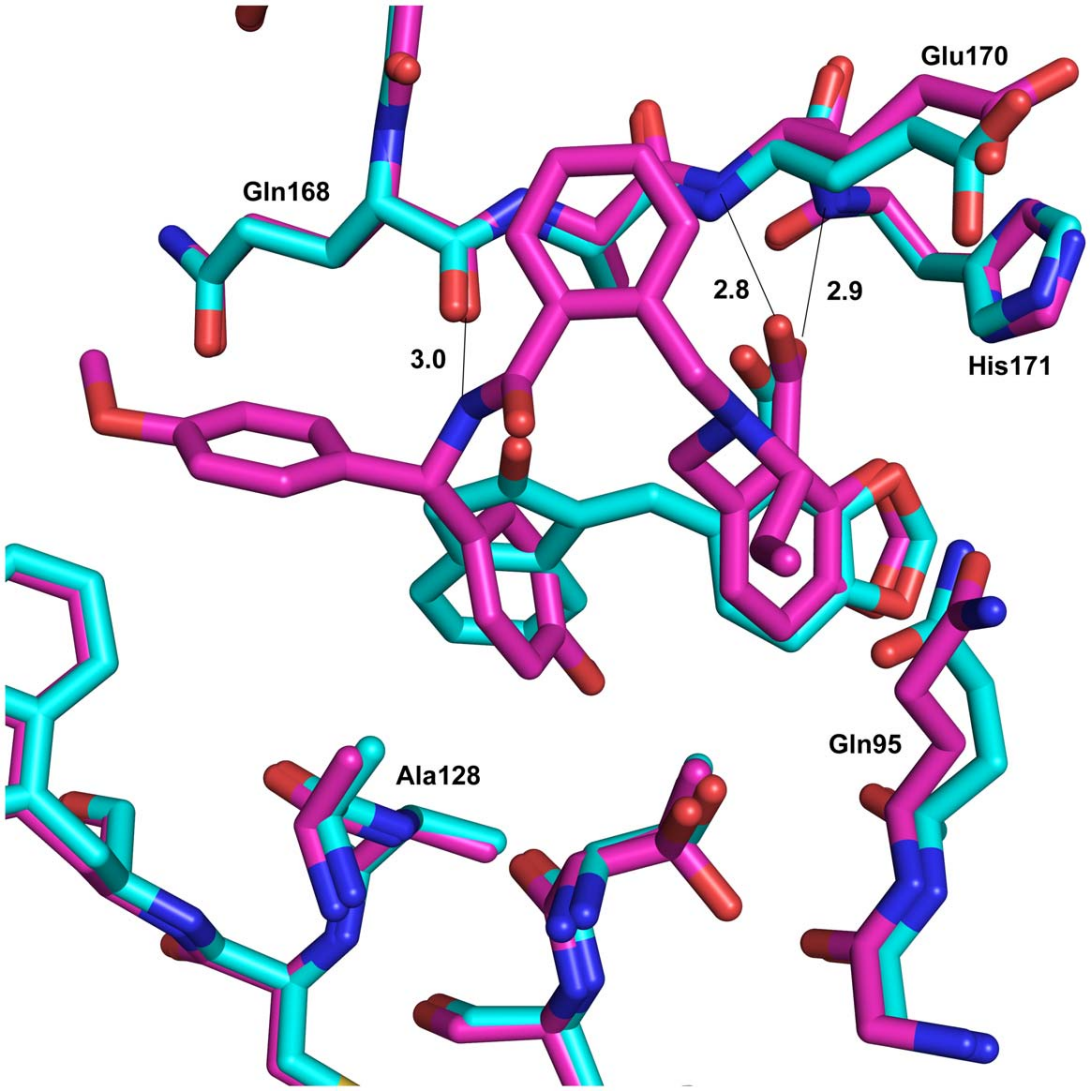
Superposition of structures with 2 (cyan) with 11 (magenta) in the LEDGF site. PDB structures 3ZT3 (**2**) and 3ZSO (**11**); an extra hydrogen bond interaction can be seen between the backbone carbonyl and the nitrogen linker in **11**. The methoxyphenyl ring of **11** also falls deeper into the hydrophobic cavity of the site.

**Figure 5:**
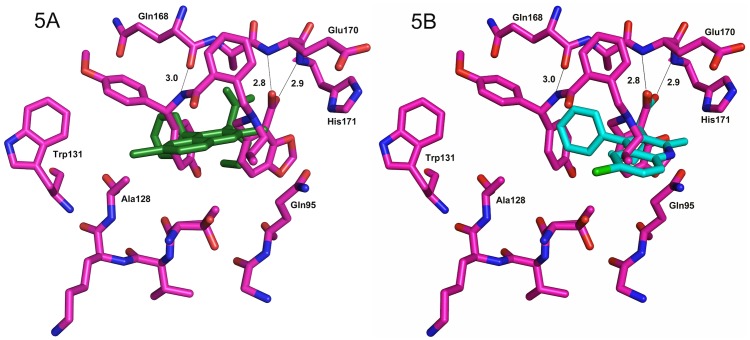
Superposition of 3LPU structure over structure with compound 11. Overlay of 3LPU structure (LEDGIN-6, CX04328, from Christ *et al*. [8]) with compound **11**. The superposition of 144 residues in common for a monomer gave a r.m.s.d. of 0.82Å overall. 5A) CX04328 is shown in dark green as seen when the proteins are superposed. 5B) CX04328 is shown in cyan when it is overlaid using the common carboxylic acid motif with **11**. The differences are due to different crystal forms making different dimers (3LPU is found as a monomer and 3ZSO as a dimer in the asymmetric unit, so a dimer for 3LPU is generated from crystallographic symmetry and 3ZSO forms a ‘biological dimer’). Dotted lines and numbers refer to the hydrogen bonds and distances (in Å) made between compound **11** and HIV integrase.

A comparison of our initial compound **2** bound in the crystal structure superposed on to the crystal structure with compound **11** can be seen in Figure 4. The amine of **11** sits deeper in the LEDGF hydrophobic pocket and makes another hydrogen bond to the HIV IN backbone (the carbonyl of Gln168). This hydrogen bond is recapitulating the bond seen in the crystal structures to the backbone amine of Ile365 of the IBD (PDB 2B4J) and of cyclic peptides (e.g. PDB 3AVB) bound to this site on HIV IN [6], [16]. Figure 5 shows a similar orientation of **11** but with the Christ *et al.* LEDGIN-6 (CX04328, PDB code 3LPU) [8] superposed in two positions. Due to the differences in crystallographic packing in the two structures (3LPU is a monomer and the structures presented here are dimers in the asymmetric unit), superposition of the protein structure (the monomer in 3LPU to one of the monomers in the dimer of 3ZSO, 144 residues align with a r.m.s.d. of ∼0.8Å) does not align the key carboxylic acid motifs of the compounds, so one alignment, 4A, is based on the superposition of the protein and the other, 4B, is based on superposition of the compounds. In both alignments, one can see that **11** delves deeper into the LEDGF pocket and makes additional hydrophobic interactions as well as the additional hydrogen bond to Gln168.

## Discussion

HIV IN represents an attractive target for drug therapy as it is required for viral DNA integration, preventing this step can reduce viral replication and viral load. Current IN inhibitors, such as raltegravir (Isentress™), bind at the catalytic site of IN and resistance is developed rapidly in the clinic even under optimized HAART [42]. We therefore set out to develop small molecule inhibitors of HIV IN that did not target the catalytic site. Our approach was to perform a fragment screen by SPR and NMR, confirming the hits in these screens by crystallography, and then screen analogues of these to obtain our first lead series.

SPR screening of the Maybridge fragment library identified benzo[d] [1], [3]dioxole-4-carboxylic acid as a hit compound. A database similarity and substructure search of the CSIRO compound library revealed analogues of the hit compound which were screened by SPR. One of these analogues, lactone **1**, was identified as a new hit. High resolution X-ray crystallographic studies showed that on soaking a solution of this compound into a crystal of HIV IN, the compound bound into two sites, one being the LEDGF site, the other being a pocket not previously described (data not shown). However, the electron density found in both sites did not closely match structure **1**, but instead closely matched the acid form **2**, which is an isomer of lactone form **1**. The acid **2** was used as a starting point for a hit-to-lead discovery program (clarification of the identity and isomerization of **1** is given in the supporting information text S1).

LEDGF has been shown to be integral for the localization of IN to the host DNA [17], [43] as well as enhancing strand transfer in *in vitro* protein assays. It has been also shown that full length LEDGF promotes tetramerization of full length HIV IN [44], which is required for the integration of both viral DNA ends into the host chromosome [45]. It has been shown that the two LEDGF binding sites present in the HIV IN dimer are only low affinity for the LEDGF binding domain (IBD), while the tetrameric form of IN presents two high affinity and two low affinity sites [15], [44]. Expression of just the IBD of LEDGF can out-compete endogenous LEDGF and lead to reduced HIV replication [46]–[48]. The equilibrium dissociation constant of the HIV IN dimer determined by Tsiang *et al* [15] was ∼65 pM, whereas the LEDGF-IN association has been calculated to be ∼10 nM. LEDGF can promote tetramer formation at concentrations of 1–14 µM HIV IN [15]. Our AS and counter screen assays were both conducted at 30°C and with 30 nM final concentration of HIV IN to give over 90% dimer and virtually no tetramer. We assumed a classical binding model in our studies as previous work [15] showed that the differences seen between using a complex model for the IN dimer and the classical model are slight.

Recent studies measuring amide proton exchange rates for the IN CCD [49] have suggested that the residues of the LEDGF binding site, in particular residues 167–173 have relatively high exchange rates, suggesting that they are solvent exposed. For ligand binding, displacement of bound water and/or stabilization of a mobile protein domain results in a change in entropy, and the observation that the LEDGF binding site has a large solvent interface [49] suggests a high energetic penalty to molecules interacting with this region of IN unless they can compensate for loss of the solvating interactions by bonds or hydrophobic interactions. Despite this, both previous work [8], [11], [12] and this current work show that small molecule inhibitors directed specifically to the LEDGF binding site of HIV IN can bind this site and disrupt the LEDGF-IN interaction. This study has identified compound **11** which was shown to interact with HIV IN in isolated protein assays, both AS and SPR, and it was confirmed that this translated to inhibition of HIV infectivity in a cell assay with an EC_50_ of 29 µM. Also, crystallographic analysis of the complex of compound **11** showed an interaction with Gln168 of the IN, demonstrating that an extended segment of 167–173 can be targeted for inhibitor design. In design of antiviral inhibitors, interactions with the viral protein backbone are advantageous, since a simple mutation by substitution of the amino acid (i.e. a change of sidechain) will not avoid the interaction.

SPR was effectively used as a fast and efficient screen of compounds which were later more fully characterised by the AS assay and X-ray crystallography. As is typical of a medicinal chemistry campaign, there is not 100% correlation between all of the assays for any given compound (see Table 1). The physical parameters of the assays are different (e.g. SPR has protein immobilised on a chip and compounds are flowed over this, whereas the AS assay and crystallography are static) as are the buffer conditions (somewhat different salts, buffers, temperature and pH are used for the three- see Materials and Methods). In addition, the SPR assay seems to be somewhat more sensitive to the solubility of the compound than the other two techniques used.

The strategy of designing compounds that bind at both the catalytic and LEDGF binding site of IN and therefore achieve inhibition of both functions has recently been proposed by two groups [26], [27]. It remains to be seen whether a possible unwanted outcome of such a strategy would be that reversible binding at one site could lead to concentrations of the compound within the cell less than the IC_90_ for the second site, with subsequent partial occupancy of that site and therefore favor resistance mutations. Our strategy was instead to use structure based design to optimize interactions at the LEDGF site without forming interactions at the catalytic site. Indeed compounds **10** and **11** were inactive in a strand transfer assay which did not contain LEDGF (data not shown), therefore we can conclude they do not bind directly at the active site of HIV IN. It was also shown that compound **11** was as active in a cell infectivity assay where the IN contained double active site mutations, either QHGS or NHEQ, compared to the WT (54, 37 and 29 µM respectively). This confirms that the inhibition by compound 11 is unaffected even in the whole cell where IN has mutations that produce resistance to inhibition by active site directed IN inhibitors.

In conclusion, we have studied the LEDGF binding pocket of HIV IN by using a series of synthetic compounds based on initial hits from a fragment based screen and have identified low micromolar inhibitors based on *in vitro* biochemical, biophysical and cellular assays. These compounds represent new scaffolds for the design of inhibitors of HIV integration.

## Supporting Information

Figure S1 **A to E:** **Fo-Fc difference density maps.** Fo-Fc difference density maps of **11** (PDB 3ZSO), **10** (PDB 3ZSQ), **8** (PDB 3ZSW), **6** (PDB 3ZSZ), **2** (PDB 3ZT3) (respectively) in the LEDGF binding pocket of HIV integrase. All figures are in approximately the same orientation and the electron density (green chicken wire) is set at 3 σ in all cases.
(TIF)

Text S1 **Information on the isomerization of compound 1 to 2.**
(DOC)
