# Correction: Small Molecule Inhibitors of the LEDGF Site of Human Immunodeficiency Virus Integrase Identified by Fragment Screening and Structure Based Design

**DOI:** 10.1371/annotation/eb238f0f-7582-4318-af95-64ac98d3b0be

**Published:** 2013-07-24

**Authors:** Thomas S. Peat, David I. Rhodes, Nick Vandegraaff, Giang Le, Jessica A. Smith, Lisa J. Clark, Eric D. Jones, Jonathan A. V. Coates, Neeranat Thienthong, Janet Newman, Olan Dolezal, Roger Mulder, John H. Ryan, G. Paul Savage, Craig L. Francis, John J. Deadman

In Table 1, the wrong PDB codes were associated with the chemical structures shown (Figure 1 and Table 1). Please use the corrected version of Table 1 at the following link:

http://www.plosone.org/corrections/pone.0040147.t001.cn.docx

