# Supplementary figures and images for: Small Molecule Inhibitors of the LEDGF Site of Human Immunodeficiency Virus Integrase Identified by Fragment Screening and Structure Based Design
Source: PLoS One. 2012 Jul 10;7(7):e40147. doi: 10.1371/journal.pone.0040147 (PMC3393750; doi:10.1371/journal.pone.0040147)

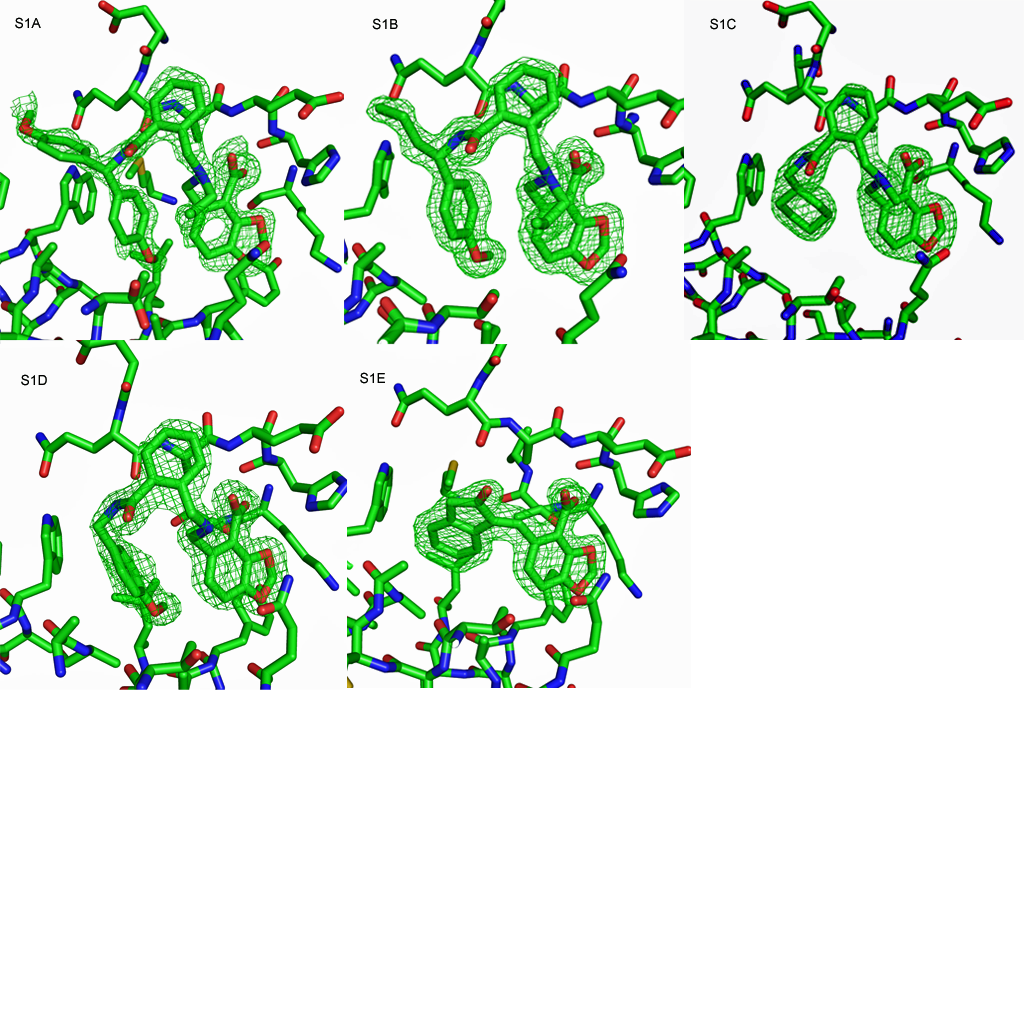

Supplement: Figure S1 — A to E: Fo-Fc difference density maps. Fo-Fc difference density maps of 11 (PDB 3ZSO), 10 (PDB 3ZSQ), 8 (PDB 3ZSW), 6 (PDB 3ZSZ), 2 (PDB 3ZT3) (respectively) in the LEDGF binding pocket of HIV integrase. All figures are in approximately the same orientation and the electron density (green chicken wire) is set at 3 σ in all cases. (TIF) [file pone.0040147.s001.tif]
