# Supplementary material for: Small Molecule Inhibitors of the LEDGF Site of Human Immunodeficiency Virus Integrase Identified by Fragment Screening and Structure Based Design
Source: PLoS One. 2012 Jul 10;7(7):e40147. doi: 10.1371/journal.pone.0040147 (PMC3393750; doi:10.1371/journal.pone.0040147)
Supplement: Text S1 — Information on the isomerization of compound 1 to 2. (DOC) [file pone.0040147.s002.doc]

**Supporting information:**

**Text S1:**

**Identity and isomerisation of compound 1**

The solid sample **1** was analysed by ^1^H NMR (400MHz, CDCl_3_) and the data obtained was consistent with the literature data [S1] and with the designated structure **1** (mixture of diastereomers). In particular, the signals at *ca.* δ 4.0 and δ 4.3 ppm (integrating to one proton) were assigned to the diastereomeric methine protons (-ArCHCO-) of the indanyl moieties for each diastereomer, and the complex multiplet at *ca.* δ 3.5 ppm (integrating to 2 protons) was assigned to the other methylene protons (-ArCH_2_CO-) of the indanyl ring systems for both diastereomers. The ^1^H NMR spectrum of a fresh solution of **1** in d_6_-DMSO showed three sets of signals at *ca.* δ 4.4, 3.6 and 3.4 ppm due to the indanyl methine and methylene protons, indicating that the archived sample, when freshly dissolved in d_6_-DMSO, was indeed the lactone structure **1** (mixture of diastereomers).

The stability of the lactone **1** in d_6_-DMSO was studied by ^1^H NMR spectroscopy. After standing for six hours at ambient temperature, the ^1^H NMR spectrum of the solution showed signals due to a *ca.* 1:4 mixture of lactone **1** and a new species. After 1 day, the ^1^H NMR spectrum of the solution showed the signals due to the lactone **1** had almost completely disappeared and had been replaced by signals of the new species. After 5 days at room temperature, there was little further change in appearance of the ^1^H NMR spectrum of the solution indicating that equilibrium had been achieved. The ^1^H NMR data for the new species within this mixture was consistent with the acid form **2**, as predominantly one isomer, presumably the *E*-isomer **2** (Scheme 1). In particular, a singlet at *ca.* δ 3.6 ppm (integrating to two protons) was assigned to the indanyl methylene protons (-ArCH_2_CO-).

These studies indicate that the reason for the acid form **2** binding to the protein in the crystallographic studies, is that, on dissolution of the lactone **1** in DMSO, equilibrium is established between the lactone **1** and the acid **2**, with the latter preferentially binding to the protein. Further work is underway in order to understand this apparent solvent-dependent isomerization and will be reported in due course.

**Scheme S1**

**Supporting Information References:**

Reference S1. Hung TV, Mooney BA, Prager RH, Tippett JM (1981) Central nervous system active compounds. VII. Phthalide synthesis by lithiation of alkoxyaromatics. Australian Journal of Chemistry 34: 383 - 395.
